# Voltage- and time-dependent valence state transition in cobalt oxide catalysts during the oxygen evolution reaction

**DOI:** 10.1038/s41467-020-15925-2

**Published:** 2020-04-24

**Authors:** Jing Zhou, Linjuan Zhang, Yu-Cheng Huang, Chung-Li Dong, Hong-Ji Lin, Chien-Te Chen, L. H. Tjeng, Zhiwei Hu

**Affiliations:** 1 Key Laboratory of Interfacial Physics and Technology, Shanghai Institute of Applied Physics, Chinese Academy of Sciences, Shanghai, 201800 China; 2 University of Chinese Academy of Sciences, Beijing, 100049 China; 3 Department of Physics, Tamkang University, 151 Yingzhuan Road, New Taipei City, 25137 Taiwan; 4 National Synchrotron Radiation Research Center, 101 Hsin-Ann Road, Hsinchu, 30076 Taiwan; 5 Max Planck Institute for Chemical Physics of Solids, Nöthnitzer Strasse 40, 01187 Dresden, Germany

**Keywords:** Electrocatalysis, Density functional theory, Electrocatalysis, Characterization and analytical techniques

## Abstract

The ability to determine the electronic structure of catalysts during electrochemical reactions is highly important for identification of the active sites and the reaction mechanism. Here we successfully applied soft X-ray spectroscopy to follow in operando the valence and spin state of the Co ions in Li_2_Co_2_O_4_ under oxygen evolution reaction (OER) conditions. We have observed that a substantial fraction of the Co ions undergo a voltage-dependent and time-dependent valence state transition from Co^3+^ to Co^4+^ accompanied by spontaneous delithiation, whereas the edge-shared Co–O network and spin state of the Co ions remain unchanged. Density functional theory calculations indicate that the highly oxidized Co^4+^ site, rather than the Co^3+^ site or the oxygen vacancy site, is mainly responsible for the high OER activity.

## Abstract (web summary)

Determining catalyst electronic structures during electrochemical reactions is crucial to understand mechanisms. Here authors perform in operando soft X-ray spectroscopy on a cobalt oxide catalyst during O_2_ evolution and observe voltage and time-dependent valence state transitions.

## Introduction

The oxygen evolution reaction (OER) plays a crucial role in many modern energy storage and conversion technologies such as solar/electricity-driven water splitting systems, rechargeable metal–air batteries, and regenerative fuel cells. However, the OER still constitutes a bottleneck in these electrochemical devices because of the intrinsically sluggish kinetics involving multistep proton-coupled electron transfer processes^1,2^. The widespread use of alkaline water electrolysis for the OER strongly depends on the availability of low-cost and efficient electrocatalysts. New materials need to be designed to replace the benchmark OER catalysts that typically consist of precious metal oxides such as IrO_2_ and RuO_2_. For this purpose, 3*d* transition-metal (TM) oxides have recently emerged as promising candidates. They are inexpensive because they are earth abundant and environmentally friendly and have tunable chemical reactivities depending on the type of electronic and crystal structures. During the past decade, many 3*d* oxides such as spinels^3,4^, perovskites^5,6^, rock salt^7^, and oxyhydroxides^8–12^, have become serious candidates for OER catalysts. The intricate interplay of charge, spin, orbital, and coordination degrees of freedom of 3*d* ions in oxides makes possible the preparation of highly active catalysts^5,6,13^.

The theoretical description of the OER involves four electron charge-transfer steps on surface metal sites. Very recently, anionic redox processes at lattice-oxygen sites were also considered^14,15^. These are complex processes, and tremendous efforts have been devoted to identifying the key parameters^5,6,12,13,16^. These parameters are usually identified based on the electronic structure of the as-prepared materials. There is, however, an increasing amount of evidence that electrocatalysts also undergo crystallographic changes under OER conditions^8,17–19^. For example, surface amorphization combined with a change in the Co–O network from corner-sharing to edge-sharing was observed for Ba_0.5_Sr_0.5_Co_0.8_Fe_0.2_O_3−*δ*_ (Ref. ^20^). Under an electric field, SrCoO_3−*δ*_ converts from the perovskite phase to brownmillerite SrCoO_2.5_ and further to the unexplored H-SrCoO_2.5_ phase^21,22^.

It is clear that in operando experimental studies are needed to elucidate the mechanism of the OER. Many particular intermediate states of 3*d* TM-oxide catalysts were identified by previous operando infrared spectroscopic studies^23–26^. The earlier infrared work identified the oxyl radical (Ti–O^•^) at the n-SrTiO_3_/aqueous interface^24^, while very recent work indicated both the oxyl radical (Ti–O^•^) that terminates the surface, and the bridged intermediate (Ti–O^•^–Ti) parallel to the surface^26^. Other operando infrared spectroscopic studies also observed the Co(IV)=O intermediate in Co_3_O_4_ with a mixed Co^2+^/Co^3+^ valence state^23^, and the Fe(IV)=O intermediate in haematite (α-Fe_2_O_3_) with an Fe^3+^ state^25^. The objective should not only be to follow the crystal structure changes, but also to record and understand the electronic structure and its modifications during the reaction. In fact, depending on how detailed and specific the information is, knowledge about the electronic structure can be most valuable for identification of the active sites. The soft X-ray absorption spectra at the 3*d* TM-L_2,3_ and O–K edges are highly sensitive to the valence state, spin state, and local environment of 3*d* TM elements. Note that the valence-state transition of a high-valent TM element does not necessarily indicate a gain of one hole in the metal 3*d* state. For instance, the Co^4+^ oxides BaCoO_3_ (Ref. ^27^) and SrCoO_3_ (Ref. ^28^) are negative charge-transfer systems, in which the 3*d*^5^ configuration has a contribution of <10%, whereas the dominant configuration is 3*d*^6^L (L stands for the hole in the ligand O 2*p* states) with *t*_2*g*_^5^*e*_*g*_^1^L_*eg*_ for a low-spin state and *t*_2*g*_^4^*e*_*g*_^2^L_*eg*_ for an intermediate-spin state as shown in Ref. ^28^, induced by very strong covalence between Co 3*d* and O 2*p*. Such O 2*p* holes can be directly studied by soft X-ray absorption spectroscopy (SXAS) at the O–K edge, especially for an edge-shared Co–O network, which presents a well-separated Co^3+^- and Co^4+^-related spectral feature in the spectra of Co^3+^/Co^4+^ mixed valence oxides^29,30^. Thus, the O–K SXAS spectra can generally be used to explore the Co^4+^ content via Co 3d–O 2*p* covalence^27,28^.

Here we apply SXAS at the Co-L_2,3_ and O–K edges to determine in operando the valence state, spin state, and local coordination of the Co ions in Li_2_Co_2_O_4_ during the OER. Li_2_Co_2_O_4_ has the best OER activity among materials with spinel structures and is quite comparable to the well-known benchmark IrO_2_^31^. The very detailed information that we were able to obtain about the Co local electronic structure allowed us to deduce that under OER conditions a substantial fraction of the Co^3+^ ions was converted into high-valent Co^4+^. This is associated with a delithiation process that also took place. Importantly, after the OER, the low-spin state of the Co ions and the edge-shared Co–O network remain unchanged. By combining these results with those obtained by density functional theory calculations, we can then infer that the Co^4+^ sites (with a dominant oxygen ligand hole ground state) are responsible for the high OER activity. Although it is experimentally challenging to separate the electrochemical liquid cell from the ultrahigh vacuum, our study shows that SXAS in operando is the spectroscopic method of choice for the investigation of TM *3d*-based catalysts since the atomic-like multiplet structures in the spectra contain highly specific information about the valence, spin, and local coordination of the *3d* ions.

## Results

### pH-dependence of the OER activity

**Fig. 1:**
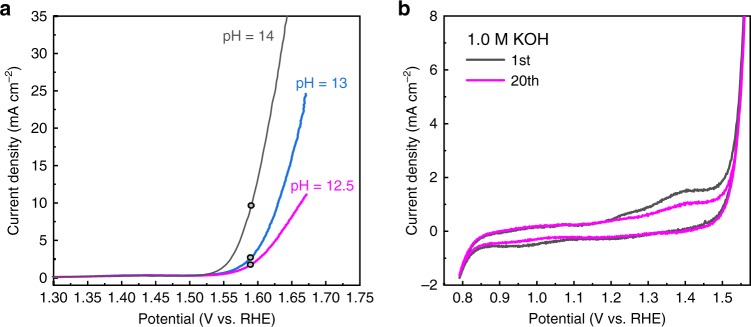
OER activity of Li_2_Co_2_O_4_. **a** Polarization curves for Li_2_Co_2_O_4_ in O_2_-saturated KOH (pH = 12.5–14) at a scan rate of 10 mV s^−1^. **b** Cyclic voltammograms of Li_2_Co_2_O_4_ loading on carbon paper at a scan rate of 5 mV s^−1^ for the 1st cycle (black) and the 20th cycle (magenta).

We started by studying the pH-dependence of the OER activity, which can provide us with an initial overview of the possible active sites and reaction paths^14,32,33^. It was suggested that a conventional OER involving four concerted proton–electron transfer steps on a surface metal-ion center, exhibited pH-independent activity, while lattice-oxygen oxidation from highly covalent oxides involving non-concerted proton–electron transfer steps exhibited a strong pH-dependent OER activity^14^. The strong pH-independent activity of Ni-based OER catalysts was attributed to the formation of negatively charged surface sites that act as OER precursors originating from a deprotonation^32^ or was associated with severe surface degradation in addition to the redox activity of lattice oxygen^33^. In the spinel oxide ZnFe_0.4_Co_1.6_O_4_, the pH-dependent OER activity was assigned as decoupled proton–electron transfers, while the lattice-oxygen contribution was excluded, considering the wide energy gap between the O p-band center and the Fermi level due to the cation-deficient spinel^34^. Figure 1a presents the OER performance of Li_2_Co_2_O_4_. It is apparent that the OER activity of Li_2_Co_2_O_4_ has a strong pH-dependence, very similar to that of ZnFe_0.4_Co_1.6_O_4_ having a Co^3.34+^ valence state but very different from that of LaCoO_3_, which contains pure Co^3+^. This is the first indication that the Co valence of Li_2_Co_2_O_4_ may have changed during the OER. We note that surface degradation as the origin of the pH-dependent OER activity can be excluded. Supplementary Fig. 1 shows, for example, that the Li_2_Co_2_O_4_ catalyst has excellent electrochemical stability, as indicated by the nearly constant overpotential over 100 h.

Our next step was to investigate the electronic structure of Li_2_Co_2_O_4_ as a function of applied voltage and time, and for this we analyzed the cyclic voltammograms to select the voltages for the SXAS experiments. Figure 1b shows the cyclic voltammograms recorded at a scan rate of 5 mV s^−1^ with Li_2_Co_2_O_4_ loading on carbon paper. We observed two redox transitions in the cyclic voltammogram: a weak transition at 1.25 V (vs. reversible hydrogen electrode (RHE)) and a relatively strong peak at 1.40 V. In CoO, Co_3_O_4_, and CoOOH, two transitions at 1.4 and 1.5 V were previously attributed to the Co^2+^/Co^3+^ and Co^3+^/Co^4+^ redox couples, respectively^4^. Since we have only a Co^3+^/Co^4+^ redox couple, the relatively strong peak at 1.4 V might correspond a sharp valence-state transition of cobalt ions from Co^3+^ to Co^4+^, while the weak feature at 1.25 V was assigned to a minor contribution from the of Co^3+^ → Co^4+^ transition, as shown in Fig. 1b. The height of the strong peak at 1.4 V decreases with an increasing number of CV scans from the 1st cycle (black) to the 20th cycle (magenta). This might suggest that the number of cobalt ions expected to undergo the valence-state transition gradually decreases in Li_2_Co_2_O_4_. Here, we observed a sharp increase in the current density at 1.55 V, where the material is activated to be a stable, highly active OER catalyst under electrochemical conditions.

### In operando SXAS studies under OER conditions

**Fig. 2:**
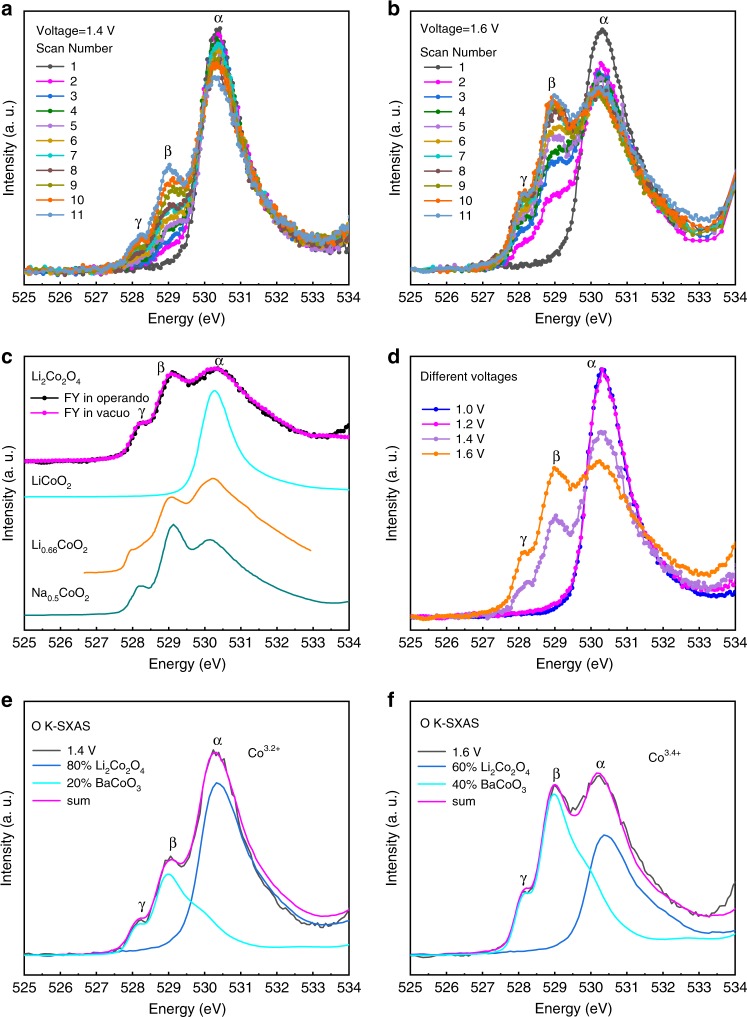
In operando O–K SXAS spectra of Li_2_Co_2_O_4_. **a**, **b** O–K SXAS spectra of Li_2_Co_2_O_4_ in operando as a function of the number of scans (2 min for each scan) at applied voltages of 1.4 and 1.6 V, respectively. **c** Comparison of the spectra during the OER (black circles, in operando) and after the OER (magenta circles, in vacuo). Also included are the spectra of LiCoO_2_ (cyan line) with the low-spin Co^3+^ state, mixed-valent Co oxide Li_0.66_CoO_2_ (orange line, from Ref. ^29^), and Na_0.5_CoO_2_ (dark cyan line, from Ref. ^30^) with the same 90° Co–O–Co bond angle. **d** Spectra of Li_2_Co_2_O_4_ at different applied voltages. **e**, **f** Experimental (black lines) and simulated (magenta lines) spectra of Li_2_Co_2_O_4_ under an applied voltage of 1.4 and 1.6 V, respectively, with the simulations being constructed as weighted sums of the spectra of the as-prepared Li_2_Co_2_O_4_ (blue) representing the low-spin Co^3+^ state and of BaCoO_3_ (cyan, from Ref. ^27^) representing the low-spin Co^4+^ configuration.

In Fig. 2a, b we present the O–K spectra of Li_2_Co_2_O_4_ in operando as a function of the number of scans (taken within 2 min for each scan) and the applied voltages. Upon applying 1.4 V, the current density is still very weak, as shown in Fig. 1b. However, one can already observe in Fig. 2a a clear increase in the intensity of two new spectral features, *β* and *γ*, with an increasing number of scans. After 10 scans (20 min), the spectral intensities of *β* and *γ* reach a maximum and remain unchanged with further scans as shown in Supplementary Fig. 2. For an applied voltage of 1.6 V, under which there is a sharp rise in the current density, as displayed in Fig. 1b, the spectral intensities of peaks *β* and *γ* increase much more quickly, as shown in Fig. 2b. The intensity increase stopped after 20 min (see Supplementary Fig. 2) but reached a much higher maximum value.

To interpret the time-dependent O–K SXAS spectra taken at 1.4 and 1.6 V, we also present for comparison in Fig. 2c the spectra of the layered LiCoO_2_ (cyan line), Li_0.66_CoO_2_ (orange line, from Ref. ^29^), and the layered Na_0.5_CoO_2_ (dark cyan line, from Ref. ^30^) having Co^3+^, Co^3.34+^, and Co^3.5+^ valence states, respectively. All three reference materials have the same low-spin state and the same edge-shared Co–O network at a 90° Co–O–Co bond angle. The absolute energy positions of all spectra were calibrated using the sharp pre-edge peak at 531.7 eV in the O–K SXAS spectrum of NiO measured simultaneously^35^. All the peaks below 533 eV represent the unoccupied O 2*p* states that are mixed into the unoccupied Co 3*d* states. The sharp single peak *α* at 530.25 eV for the as-prepared Li_2_Co_2_O_4_ in Fig. 2a, b, as well as that for LiCoO_2_ in Fig. 2c, reflects a fully occupied *t*_2*g*_^6^ state associated with the low-spin Co^3+^ ion^29^. In this case, only transitions to the unoccupied *e*_*g*_ states are possible. For the mixed-valent Co oxides Li_0.66_CoO_2_ (from Ref. ^29^) and Na_0.5_CoO_2_ (from Ref. ^30^), two additional peaks, *β* and *γ*, appear at lower energies. They originate from transitions to the unoccupied *t*_2*g*_ and *e*_*g*_ orbitals of the Co^4+^ ion, respectively, as also observed in BaCoO_3_ (see Fig. 2e, f (blue line)), also having the low-spin state and the same 90° Co–O–Co bond angle. Thus, the increase in the spectral intensities of the *β* and *γ* features during the OER indicates an increase in the Co valence state. In other words, there is a transition from a Co^3+^ state to a Co^4+^ state in part of the Li_2_Co_2_O_4_ catalyst during the electrochemical reaction. The spectra of Li_2_Co_2_O_4_ at different applied voltages are presented in Fig. 2d, where one can observe only a negligible amount of Co^4+^ ions at 1.2 V. The spectral intensity of Co^4+^ (*β* and *γ*) in Li_2_Co_2_O_4_ after 20 min under an applied voltage of 1.4 V is only slightly smaller than that of Na_0.75_CoO_2_ in the Co^3.25+^ state^30^, while after 20 min under 1.6 V, it lies between those of Li_0.66_CoO_2_ and Na_0.5_CoO_2_. The Co valence in these two cases can be estimated by constructing a weighted sum of the Li_2_Co_2_O_4_ spectrum (blue line) and BaCoO_3_ spectrum (cyan line, from Ref. ^27^), as shown in Fig. 2e, f. We find Co^3.2+^ and Co^3.4+^ valences for these two cases, respectively.

**Fig. 3:**
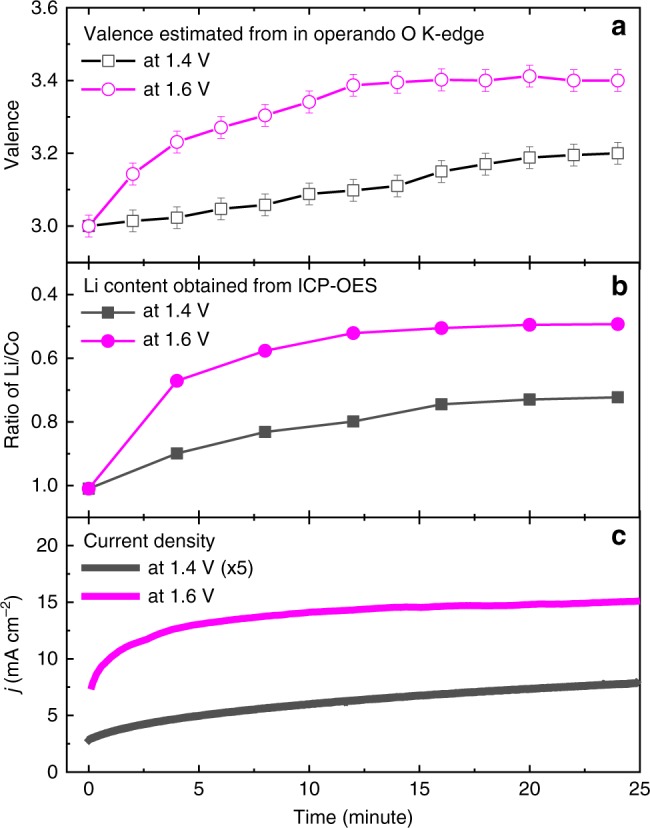
Evolution of Li_2_Co_2_O_4_ under applied voltages of 1.4 and 1.6 V over time. **a** Change in the Co valence determined from in operando SXAS, **b** evolution of the lithium/cobalt ratio, **c** evolution of current density *j*.

Using the same procedure as shown in Fig. 2e, f, we extract and display in Fig. 3a the time evolution of the Co valence under applied voltages of 1.4 (black open squares) and 1.6 V (magenta open circles). In Fig. 3b and Supplementary Table 1, we show the lithium content as a function of time. The data sets reveal that the composition of the as-prepared material, i.e., at 0 min in Fig. 3b, is in accordance with the chemical formula of Li_2_Co_2_O_4_, and after 20 min of the OER at an applied voltage of 1.6 V, half of the lithium ions were removed. The current density *j* (solid line), as shown in Fig. 3c, also has a similar trend with time. This strongly indicates that the OER activity is directly related to the appearance of Co^4+^ ions.

We have carried out a number of verification experiments to ensure that our experimental observations are correct. We also carried out O–K SXAS measurements under ultrahigh vacuum conditions. In Fig. 2c we show the in vacuo spectrum of Li_2_Co_2_O_4_ after 20 min of OER under an applied voltage of 1.6 V (magenta circles). A comparison with the in operando spectrum (black circles) reveals that the spectra are identical in the energy region of interest. There are differences between the in vacuo and in operando spectra, but these occur only in the energy region above 533 eV. As illustrated in Supplementary Fig. 3, there is extra background in the in operando spectrum above 533 eV, which can be attributed to the oxygen in the electrolyte. Thus, from the relevant features that appear below 533 eV, we can safely conclude that the O–K SXAS experiments (in operando and in vacuo) establish the irreversible valence change of Co during the electrochemical reaction. Next, we also performed SXAS experiments at the Co-L_2,3_ edges. In Supplementary Fig. 4e, we show that there is a change and shift to higher energies of the Co spectrum when comparing the Li_2_Co_2_O_4_ after the OER with the as-prepared Li_2_Co_2_O_4_, indicating an increase in the Co valence^27,36,37^. We also quantitatively analyzed the Co-L_2,3_ spectra in terms of a weighted superposition of the theoretical LS Co^3+^ and LS Co^4+^ spectra, see Supplementary Fig. 5a. We found 60% Co^3+^ and 40% Co^4+^, i.e., an LS Co^3.4+^ state, which is fully consistent with the O–K data. We note that the sharp low-energy shoulder S at 779.7 eV in the Co-L_2,3_ spectra as displayed in Supplementary Figs. 4e and 5a is a transition to the unoccupied *t*_2*g*_ and a fingerprint of the LS Co^4+^ state, as observed in the BaCoO_3_ spectrum (wine) in Supplementary Fig. 4a. As a final check, we also measured the Co-L_2,3_ and O–K spectra by the more surface-sensitive total electron yield (TEY) method for comparison with the total fluorescence yield (TFY) spectra. Supplementary Fig. 5a, b reveals that the Co-L_2,3_ spectra are very similar and thus also extracted the Co valence, i.e., Co^3.35+^ in TEY versus Co^3.4+^ in TFY. Supplementary Fig. 6b, c shows that the relative spectral weight of the *β* and *γ* features in the O–K SXAS is also only slightly smaller in the TEY than in the TFY, fully consistent with the Co-L_2,3_ findings. We note that these small differences between the TEY and TFY spectra may suggest that part of the Co^4+^ ions at the surface are somewhat destabilized by the in vacuo conditions under which the TEY spectra were recorded. From all these checks we can safely conclude that the SXAS experiments firmly establish the irreversible change in the Co valence during the electrochemical reaction.

### Structure and morphology characterization

**Fig. 4:**
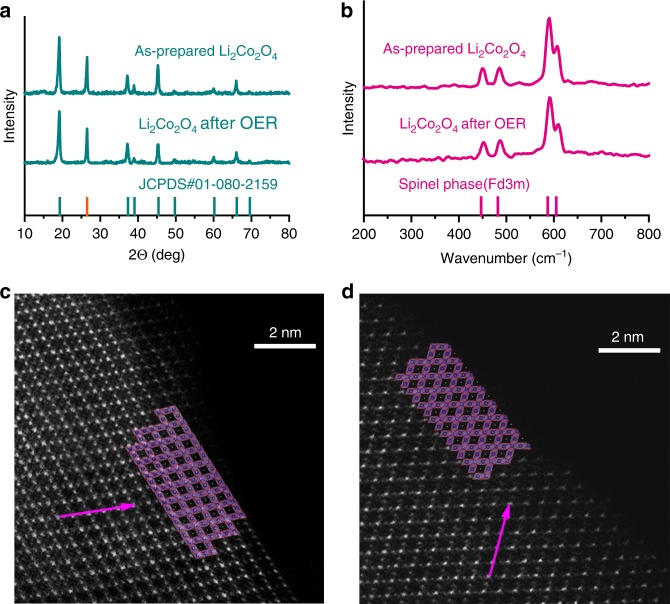
Structural characterizations of Li_2_Co_2_O_4_. **a** XRD patterns of Li_2_Co_2_O_4_ as prepared and after OER. The orange line corresponds to the XRD peak of the carbon fiber paper. **b** Raman spectra of Li_2_Co_2_O_4_ as prepared and after OER. **c** HAADF-STEM images of as-prepared Li_2_Co_2_O_4_. **d** HAADF-STEM images of Li_2_Co_2_O_4_ after OER. Both are viewed down the [110] zone axis, and the arrows are along the [1-11] direction.

We now investigate the effect of OER on the crystal structure of Li_2_Co_2_O_4_ using X-ray powder diffraction (XRD). Figure 4a shows the data of the sample as prepared (top) and after the OER (middle) under an applied voltage of 1.6 V for 20 min in an O_2_-saturated 1 M KOH aqueous solution. The XRD patterns can be indexed by the standard Fd-3m symmetry (JCPDS No. 01-080-2159) corresponding to a pure spinel structure. We also observe that the patterns before and after the OER are essentially identical and that there are no additional peaks that otherwise may indicate the presence of an impurity phase. We note that there is a reflection from the carbon paper substrate, which we have indicated by the orange line at the bottom of Fig. 4a. The results demonstrate that the overall crystalline phase of Li_2_Co_2_O_4_ remains unchanged after the OER. We can rule out impurity phases such as Co_3_O_4_, which was reported previously after the electrochemical process^38^. To further confirm our point, we have studied the Raman spectrum, which is also highly sensitive to impurity phases. As shown in Fig. 4b, the Raman spectra of Li_2_Co_2_O_4_ before and after the OER display four peaks at 450, 487, 590, and 608 cm^−1^, corresponding to the *A*_1*g*_ + *E*_*g*_ + 2*F*_2*g*_ active modes, indicating again a pure spinel structure for the sample both before and after the OER.

As a next step, we examined the atomic arrangement in the Li_2_Co_2_O_4_ nanoparticles in the near surface regions using a high-angle annular-dark-field scanning transmission microscope (HAADF-STEM). Figure 4c, d shows the HAADF-STEM images viewed down the [110] axis of the Li_2_Co_2_O_4_ samples before and after the OER, respectively. We have observed that the atomic arrangement in the as-prepared nanoparticles matches very well the spinel lattice with the space group Fd-3m and that Co ions and Li ions occupy the 16*d* and 16*c* octahedral sites, respectively. The HAADF -STEM images show well-defined lattice fringes in the surface region of Li_2_Co_2_O_4_ samples, revealing no change in the crystal structure at the surface of Li_2_Co_2_O_4_ due to the OER. From the combination of SXAS, XRD, and HAADF-STEM analysis, we can infer that the low-spin state of the Co ions remains unchanged. We can also conclude that there is no complete change from the edge-shared network to corner-shared network, since the O–K SXAS spectrum of perovskite (La_1−*x*_Sr_*x*_CoO_3_) is very different from that of spinel structure (Li_*x*_CoO_2_), as shown in Supplementary Fig. 7. The strong inter-site hopping smears the difference in the spectral features in the O–K edge between Co^3+^ and Co^4+^ for a corner-shared network in perovskite La_1−*x*_Sr_x_CoO_3_, while the differences between Co^3+^ and Co^4+^ are clearly seen in the spinel structure Li_*x*_CoO_2_ (Ref. ^29^) and Na_*x*_CoO_2_ (Ref. ^30^) with an edge-shared network. However, we cannot exclude slight changes in the Co–O–Co bond angle. The detailed structural changes within the data presented could be revealed in the future by theoretical studies and operando XRD experiments.

### DFT calculations for OER mechanisms and active sites

**Fig. 5:**
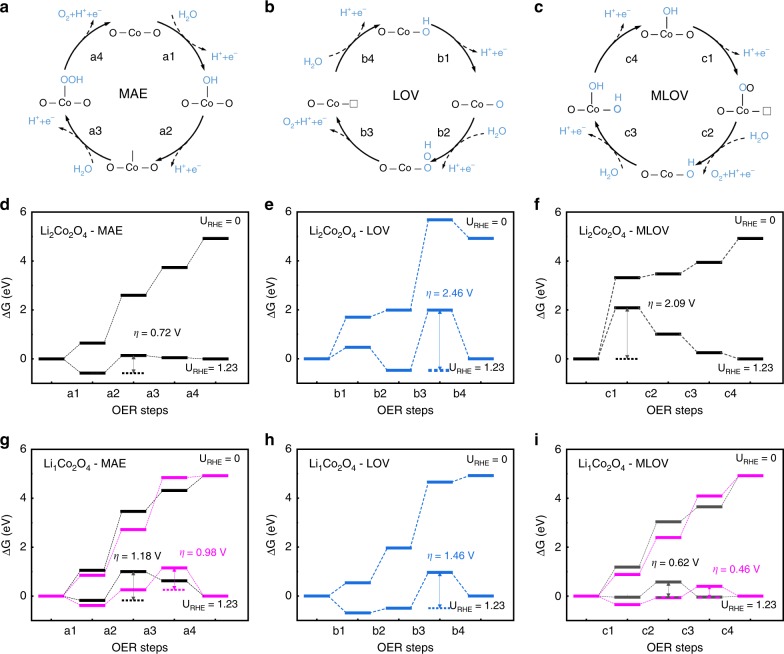
Schematic OER mechanisms involving four concerted proton–electron transfer steps. **a**, **d**, **g** MAE: this is a conventional OER mechanism, in which adsorbates are located at the transition-metal site. **b**, **e**, **h** LOV: in this scenario, adsorbates are only located at the site of the lattice-oxygen vacancy generated during redox reactions. **c**, **f**, **i** MLOV: in this scenario, adsorbates are located at both the transition-metal site and the oxygen vacancy site. The free energies under different potentials *U*_RHE_ for the OER on Li_2_Co_2_O_4_ in **d**–**f** and Li_1_Co_2_O_4_ in **g**–**i**. The black lines and the magenta lines indicate the Co^3+^ site and Co^4+^ site, respectively.

Having determined the valence state of the Co ions and the crystal structure under the OER condition, we now investigate the underlying mechanism and the active site for the OER performance in Li_2_Co_2_O_4_. For this purpose, we performed DFT calculations to model the reaction pathways. Three scenarios of the reaction mechanism were considered, as shown in Fig. 5a–c, and each involves four proton–electron transfer steps. The first one, shown in Fig. 5a, is the metal-site adsorbate evolution mechanism (MAE). This is the conventional adsorbate evolution mechanism^12^, which considers only the redox activity of the TM sites. The second one, shown in Fig. 5b, is the lattice-oxygen-vacancy-site mechanism (LOV). This mechanism was proposed very recently^14^, in which the adsorbates are located at the sites of the lattice-oxygen vacancy. The last one, shown in Fig. 5c, is the metal-and-lattice-oxygen-vacancy-site mechanism (MLOV). Here, we consider the adsorbates to be at both TM sites with different valence states and lattice-oxygen vacancy sites^15^. Both LOV and MLOV scenarios consider the role of the lattice-oxygen vacancies at the surface. However, the effect of the metal sites with a high oxidation state generated during the OER is explicitly taken into account in the MLOV scenario, while in the LOV scenario, the occurrence of a high oxidation state is merely considered the cause for the formation of the oxygen vacancies. We built models for Li_2_Co_2_O_4_ and Li_1_Co_2_O_4_ to reveal the effects of the occurrence of the Co^4+^ state generated during the OER, as shown in Supplementary Fig. 10, and calculated the free energies of the electrochemical OER process.

For Li_2_Co_2_O_4_, the Bader charge (*q*) of all Co sites at the surface is +1.47, representing Co^3+^ according to previous theoretical work^12^, while for mixed-valent Li_1_Co_2_O_4_, there are two different Co sites on the surface: the Co^3+^ site (*q* = +1.49) and the Co^4+^ site (*q* = +1.60)^12^. Figure 5d–i shows the free-energy diagrams under two different potentials *U* (*U* = 0 and 1.23 V vs. RHE) of Li_2_Co_2_O_4_ and Li_1_Co_2_O_4_ obtained from the three abovementioned OER mechanisms. The black lines and the magenta lines represent the Co^3+^ site and Co^4+^ site, respectively in Fig. 5d–i. For Li_2_Co_2_O_4_, the MAE route has the lowest overpotential (*η*) of 0.72 V among the three scenarios. The rate-limiting step is the formation of *O in this scenario. The theoretical potentials for LOV and MLOV are 2.46 and 2.09 V, respectively, which are much higher than that of MAE. The rate-limiting steps of both cases are related to the formation of oxygen vacancies. Thus, we can conclude that the stable lattice oxygen for Co^3+^ oxide limits the formation of oxygen vacancies and that the contribution of the lattice-oxygen redox reaction to OER activity is less important for as-prepared Co^3+^ Li_2_Co_2_O_4_; therefore, the OER activity mainly originates from the MAE route for the Co^3+^ state in the lattice.

In fact, during the OER process, part of the Co^3+^ ions are changed to Co^4+^ ions accompanied by delithiation, as found above. We turn to the delithiation Li_1_Co_2_O_4_. The negative charge-transfer energy of Co^4+^ ions in Co oxides leads to the appearance of unstable O 2*p* holes^39^ and favors the formation of oxygen vacancies. The calculated overpotential for Li_1_Co_2_O_4_ in the MLOV case is 0.46 V, lower than the values of 0.98 V in the MAE scenario and 1.46 V in the LOV scenario, as shown in Fig. 5g–i. In the MLOV route, the Gibbs free-energy difference (Δ*G*) of step 1 related to the formation of oxygen vacancies is much lower for Li_1_Co_2_O_4_ than for Li_2_Co_2_O_4_; thus, the rate-limiting step is changed to the adsorption of *OH in MLOV.

For Li_1_Co_2_O_4_, the ΔG of step 1 for Co^4+^ is ~0.3 eV lower than that for Co^3+^ in the MLOV route, indicating again that the lattice oxygen around Co^4+^ is very unstable and is easily removed from the lattice, forming *OO intermediates. The different rate-limiting steps between Co^3+^ and Co^4+^ also originate from the different binding energies of *OH. It is worth noting that although both the LOV and MLOV scenarios have considered the effects of the oxygen vacancy, the calculated total overpotential for LOV in Li_1_Co_2_O_4_ is much higher than that for MLOV, indicating the important role of the metal Co^4+^ site in the OER activity. If we replace distinguishable Co^3+^ and Co^4+^ with an average Co valence state, the LOV scenario is more favorable than the MAE route, as found previously^14^. The occurrence of high-valent Co^4+^ ions in the Co oxide leads to an increase in the O 2*p* band center, whose positive effects on the OER activity were well studied previously^14,40^.

## Discussion

The OER activity of Li_2_Co_2_O_4_ and many other Co oxide systems was attributed to the presence of Co ions which have their *e*_*g*_ shell occupied by an electron^41^. Starting with an LS Co^3+^ state in the as-prepared material, it was proposed that the *e*_*g*_ shell acquires an electron by considering the formation of a CoO_5_ pyramidal coordination due to the loss of oxygen at the sample surface. We found no evidence for this in our experiments. First, we would like to point out that a loss of oxygen should decrease the valence of the Co ions; i.e., part of the Co^3+^ should be converted into Co^2+^. However, our Co-L_2,3_ spectra of the Li_2_Co_2_O_4_ catalyst material before and after the OER do not show features that otherwise could have indicated the presence of Co^2+^ species. As shown in Supplementary Fig. 4f, a Co^2+^ ion would have a sharp peak at 777.8 eV, which is totally absent in our Li_2_Co_2_O_4_ spectra both before and after the OER. In addition, our DFT calculations indicate that oxygen vacancies under OER conditions are very unstable and that they quickly become filled by OH. Second, if the Co ion remains in the trivalent state when the CoO_5_ pyramidal coordination is formed, then there will be not one electron transferred to the *e*_*g*_ shell but two electrons. The associated high-spin (HS) state for the *t*_2*g*_^4^*e*_*g*_^2^ configuration is well documented in the literature for Co^3+^ ions with pyramidal local coordination^42–46^. The spectrum of such an HS CoO_5_ pyramidal system is given in Supplementary Fig. 4d and is very different from our Li_2_Co_2_O_4_ spectra. We thus can rule out the presence of *e*_*g*_ electrons in our catalyst material before and after the OER.

Our in operando and in vacuo SXAS spectra instead showed a pure LS Co^3+^ state in Li_2_Co_2_O_4_ before the OER and that it is converted to an LS Co^3.4+^ state after the OER. This is also reconfirmed by comparing the spectra to those of known Co compounds in the literature. Supplementary Fig. 4c shows that the spectrum of LiCoO_2_, which is an LS Co^3+^ system, is identical to that of the as-prepared Li_2_Co_2_O_4_. Supplementary Fig. 4b displays that the spectrum of Li_0.66_CoO_2_, which is an LS Co^3.34+^ material, is very similar to that of Li_2_Co_2_O_4_ after the OER.

Our DFT calculations indicate that the Co^4+^ site spontaneously created under OER conditions is mainly responsible for the high OER activity. It is well known that the O 2*p* to Co 3*d* charge-transfer energy Δ (defined as the energy difference between the center O 2*p* band and the upper-Hubbard band (UHB), as shown in Supplementary Fig. 11) decreases with increasing valence state of the TM ion^47^. For Co^2+^, it is positive^48,49^; for Co^3+^, it is close to zero^50^; and for Co^4+^, it can be negative^27,28,51^. Consequently, the covalency increases, i.e., from rather ionic for Co^2+^, to highly covalent for Co^3+^, and further to Co^4+^, in which the holes mainly reside on the oxygen ligands rather than on the Co^27,28^. This trend can experimentally be directly observed in the O–K SXAS spectra: Supplementary Fig. 6 shows the spectral changes upon converting CoO to the as-prepared Li_2_Co_2_O_4_, Li_x_CoO_2_, and Li_2_Co_2_O_4_ after the OER and finally to BaCoO_3_. The figure displays how the spectral weight is shifting quickly to lower energies with increasing valence. The schematic density of states of Co^3+^ and Co^4+^ oxides (in the limit of no metal-ligand hybridization) is also shown in Supplementary Fig. 11 for Co^3+^ and Co^4+^ oxides. The relation between the charge-transfer energies Δ_4+_ and Δ_3+_ is Δ_4+_ = Δ_3+_ − *U* + *δ*, where *U* is the on-site Co 3*d* Coulomb repulsion and *δ* is an extra gain originating from the lattice relaxation. It was previously found that the shift of the valence band center toward *E*_*f*_ upon converting Co^2+^/Co^3+^ to the mixed Co^3+^/Co^4+^ oxide can easily facilitate the formation of O–O^5,13,40,52,53^.

Therefore, for Co^4+^ oxides, we have high unoccupied density states with O 2*p* ligand character close to the chemical potential in the ground state^28^, which is generally denoted as the ligand oxygen hole (LOH)^27,28^. Thus, the high OER activity of the electrochemical catalysts is intimately linked to the presence of large numbers of LOHs.

Nevertheless, one must also realize that an increase in the numbers of LOHs will make gradually the material unstable^54^. It is well known that Co^4+^ oxides such as SrCoO_3_ and Sr_2_CoO_4_ can easily decompose or become off-stoichiometric, even in air; in particular, *x* cannot be reduced to zero for the Li_*x*_CoO_2_ and Na_*x*_CoO_2_ systems. We therefore suggest that perhaps a valence state of the Co ion in the range of +3.3 – +3.4 would be a good starting point to find Co oxide materials that are highly OER active and yet also structurally stable. La_0.6_Sr_0.4_Co_0.2_Fe_0.8_O_2.9_ (Ref. ^55^), SrCoO_2.7_ (Ref. ^15^), Pr_0.5_Ba_0.5_CoO_3−δ_ (Ref. ^40^), ZnFe_0.4_Co_1.6_O_4_ (Ref. ^34^), and Ba_4_Sr_4_(Co_0.8_Fe_0.2_)_4_O_15_ (Ref. ^56^) may serve as examples, together with LiCoO_2_ (Ref. ^38^) and Li_2_Co_2_O_4_, which are delithiated under OER conditions.

In summary, the origin of the high OER activity of the Li_2_Co_2_O_4_ catalyst was revealed by our in operando SXAS measurements as a function of applied voltage and time. We have observed that an irreversible valence-state transition from Co^3+^ to Co^3.4+^ occurs under OER conditions. This transition is accompanied by a delithiation process. The low-spin state of the Co ions, however, remains unchanged. No complete change from the edge-shared network to the corner-shared network can be observed. The underlying mechanism of the OER activity was then revealed by DFT calculations, which indicated that the adsorbates located at the Co^4+^ sites have a lower overall overpotential for the OER process than those located at the Co^3+^ sites or oxygen vacancy sites. The microscopic origin is the dominant O 2*p* hole characteristic of the ground state of Co^4+^ having a negative charge-transfer energy. In such cases, the O 2*p* hole is due to the strong Co 3*d*–O 2*p* covalence, which is different from the strong O 2*p*–O 2*p* bond occurring in peroxo-like (O_2_)^*n*−^ species. We suggest that the ideal valence state of the Co ions for high OER activity is Co^3.3+^–Co^3.4+^. This can be either prepared or spontaneously created under OER conditions. A higher content of Co^4+^ may increase the activity but will also lead to a decrease in the structural stability of the catalyst material.

## Methods

### Synthesis

To obtain a very small average size of the particles that is suitable for in operando soft X-ray absorption experiments, Li_2_Co_2_O_4_ was prepared by the citrate sol–gel method as follows: lithium acetate (≥99.9%, Alfa Aesar), cobalt acetate hexahydrate (≥99.9%, Alfa Aesar), citrate (≥99.5%, Aladdin), and urea (≥99%, Aladdin) were dissolved in distilled water with a mole ratio of 1:1:2:2. The solution was evaporated at 80 °C with magnetic stirring for 4 h and then transferred to an oven at 180 °C overnight to obtain a gel. Afterwards, the gel was calcined at 350 °C for 48 h to obtain Li_2_Co_2_O_4_.

### Physicochemical characterization

The structure and phases of Li_2_Co_2_O_4_ before and after the OER were identified using grazing incidence X-ray diffraction (XRD, Bruker D8-Advance AXS diffractometer with Cu K_*α*_ irradiation and an incidence angle of 1°) and Raman (Horiba XploRA confocal Raman Microscope with laser wavelength of 532 nm). It is worth noting that the laser power for the Raman experiment was only 0.2 mW to avoid a structure phase transition caused by the laser. The morphology of the samples was characterized by transmission electron microscopy (TEM, FEI Tecnai G2 F20 S-TWIN) and scanning transmission electron microscopy (STEM, FEI aberration corrected Titan G2 60–300 with convergence angle of ~21.4 mrad and camera length of ~145 mm). The elemental compositions were measured by inductively coupled plasma-optical emission spectrometry (Spectro Arcos SOP).

### Electrochemical measurements at home institute

Electrochemical measurements were conducted using an electrochemical workstation (Metrohm Autolab PGSTAT 302N) with a standard three-electrode electrochemical cell. The catalysts loaded on carbon paper with a loading mass of 0.3 mg cm^−2^ via drop-casting, Hg/HgO (1 M KOH) and Pt foil acted as the working, reference and counter electrodes, respectively. All electrochemical experiments were performed in freshly prepared O_2_-saturated KOH (pH = 12.5–14; 99.99%, Alfa Aesar). Polarization curves for Li_2_Co_2_O_4_ were recorded at a scan rate of 10 mV s^−1^, and cyclic voltammograms were recorded at a scan rate of 5 mV s^−1^. Unless specifically mentioned, all electrode potentials used in this study were referenced to the RHE and iR-corrected to compensate for the effect of solution resistance.

### Soft X-ray absorption spectroscopy

The in operando SXAS experiments at the O–K and Co-L_2,3_ edges were carried out at the 11A beam line of the National Synchrotron Radiation Research Center in Taiwan using the TFY mode. NiO and CoO single crystals purchased from Matek Material Technologie & Kristalle GmbH were recorded simultaneously in a separate ultrahigh vacuum chamber in TEY mode to serve as relative energy calibration for the measurements at the O–K and Co-L_2,3_ edges, respectively. The SXAS spectra of the as-prepared Li_2_Co_2_O_4_ and Li_2_Co_2_O_4_ after the OER were measured using both TEY and TFY modes simultaneously. A correction for the self-absorption effects in the TFY spectra was applied^57,58^. There are several reasons why we performed the operando time-dependent TFY SXAS only at the O–K edge. First, in the conversion from Co^3+^ to Co^4+^, the created holes mainly reside in the O 2*p* states; therefore, the O–K SXAS spectrum is more sensitive to them than the Co-L_2,3_ SXAS. Second, the O–K SXAS spectrum for an edge-shared network presents separated the features for Co^3+^ and Co^4+^ as well as *t*_2*g*_- and *e*_*g*_-related holes well, while the increase in Co valence mainly produced a slight energy shift in the TFY Co-L_2,3_ SXAS. Finally, in terms of practicality, each Co-L_2,3_ spectrum with reasonable statistics took more than 20 min, which is too slow to detect the time-dependent effects for the operando experiments.

For SXAS measurements, the Li_2_Co_2_O_4_ catalyst powder was dispersed in ethanol and deionized water and then sonicated for 30 min. The ink was then drop-cast on carbon paper with a loading mass of 0.3 mg cm^−2^ for ex situ SXAS experiments. For in operando experiments, the ink was dropped into the thin membrane window (100 nm silicon nitride with a 1 × 1 mm^2^ area coated by 3 nm Ti/10 nm Au from Silson Ltd) with a loading mass of ~1 mg cm^−2^. This window was used as the working electrode and to separate the liquid and the ultrahigh vacuum environment. The in operando SXAS experiments were performed using an in situ electrochemical liquid cell^59,60^ equipped with three electrodes (working, reference, and counter electrodes) under control by a VersaSTAT 3 potentiostat from Princeton Applied Research. Two platinum wires were used as the reference and counter electrodes. Here, we selected a Pt pseudoreference electrode due to space constrictions in the electrochemical cell and calibrated the potential to RHE following the procedure described by Kasem and Jones^61^. Freshly prepared O_2_-saturated 1.0 M KOH was used as the electrolyte, and the electrochemical liquid cell system also contained a liquid pump, an inlet, and an outlet tube for the electrolyte flow.

TFY was used as the detection method for the absorption signal in the in operando SXAS experiments. A photon escape depth of ~200 nm is sufficiently large to overcome the liquid region and the membrane separating the liquid from the ultrahigh vacuum. The particle size and distribution of Li_2_Co_2_O_4_ nanoparticles used for the SXAS experiments were determined by high-resolution transmission electron microscopy in Supplementary Fig. 8. The average size of particles was <20 nm, which can ensure sensitivity to the surface region of the catalyst material for SXAS measurements. Assuming that the active region for the OER reaction is within a depth of ~5 nm from the surface^14,62^, it can be estimated that ~80% of the TFY signal originates from this region. This is illustrated in Supplementary Fig. 9, which shows how the ratio of the surface-to-bulk contribution in the TFY gradually increases as the size of particles decreases.

### Density function theory calculations

All DFT calculations were performed with the Vienna Ab initio Simulation Package^63,64^ using projector-augmented wave pseudopotentials and the Perdew–Burke–Ernzenhof^65^ exchange correlation functional. The energy cutoff of the plane wave was 500 eV. To describe the strong correlation of the localized Co 3*d* states, the Hubbard *U* model^66^ was applied, and the value of *U*_eff_ (=*U* − *J*) was set to 3.52 eV according to previous work^12^. The experimental lattice constants were adopted, and the geometries were relaxed until a maximum threshold force of 0.02 eV/Å was fulfilled. For computational efficiency, a 3 × 3 × 1 Monkhorst–Pack k-point mesh was used for all calculations. The systems were initiated with Co atoms in a ferromagnetic configuration, which was allowed to evolve during the calculations. A 1 × 1 primitive cell (lattice constants are 7.9825 Å) was employed to build periodic slab models, with four Co sites per surface. There were eight atomic layers in the slab models, and four layers at the bottom were fixed during the relaxation. The thickness of vacuum spacing perpendicular to the surface was ~20 Å to prevent spurious interactions.

The overpotentials for Li_2_Co_2_O_4_ surfaces were calculated considering three scenarios of the reaction mechanism^12,14,15^. Elementary steps and all the corresponding equations considered for three scenarios of the reaction mechanism are provided in the supplementary information. The Gibbs free-energy differences were calculated using the computational hydrogen electrode model under standard conditions^12^. We considered voltage applied *U*_RHE_ = 0 and 1.23 V. The zero-point energy (ZPE) and entropy corrections were calculated according to the literature^12,15^ (Supplementary Table 2). The experimental Gibbs free formation energies and entropic contributions of H_2_O and H_2_ under standard conditions (*T* = 298 K and *P* = 1 bar) were obtained from the CRC Hanbook^67^. For the adsorbed species, the ZPEs were calculated for the (001) Li_2_Co_2_O_4_ surface. The entropy corrections for the adsorbent on the surface were considered zero, since the main contribution to the entropy is due to the translational entropy. The theoretical overpotential (*η*) was defined from the Gibbs free-energy differences at each step.

1 $$\eta = \max \left[ {\Delta {{G}}_i} \right]/{{e}} - 1.23\,{\mathrm{V}}.$$

## Supplementary information

Supplementary Information

## Data Availability

The data that support the findings of this study are available from the corresponding authors upon request. The source data underlying Figs. 1–3, 4a, b, 5d–i and Supplementary Figs. 1–7, 8b, and 9 are provided as a Source Data file.

## Source data

Source Data
