# Supplementary material for: Voltage- and time-dependent valence state transition in cobalt oxide catalysts during the oxygen evolution reaction
Source: Nat Commun. 2020 Apr 24;11:1984. doi: 10.1038/s41467-020-15925-2 (PMC7181785; doi:10.1038/s41467-020-15925-2)
Supplement: Supplementary file 1 — Supplementary Information [file 41467_2020_15925_MOESM1_ESM.pdf]

Supplementary Information

Voltage- and time-dependent valence state transition in cobalt oxide catalysts during the oxygen evolution reaction

Jing Zhou et al.

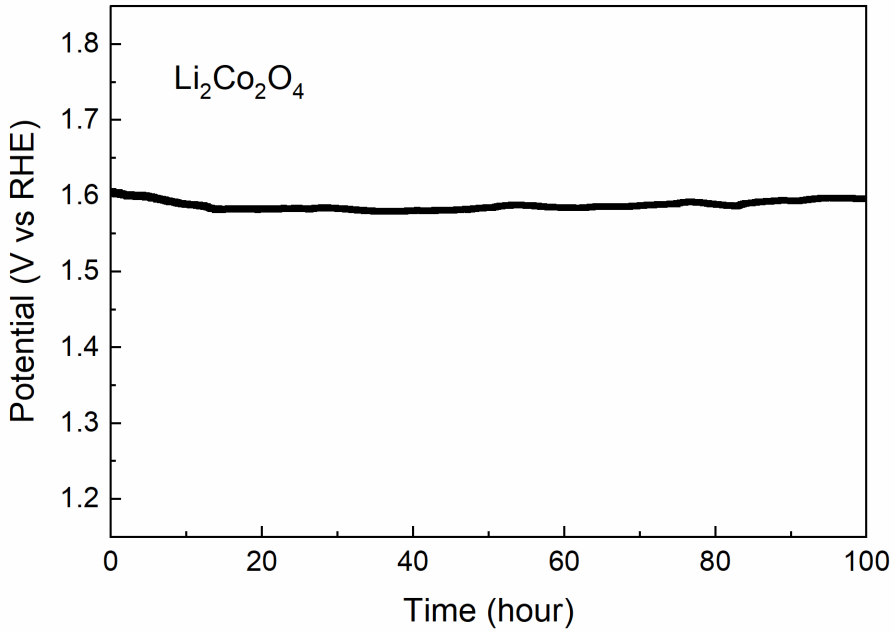

Supplementary Figure 1. Galvanostatic stability of $Li_2Co_2O_4$ at a geometric current density of 10 mA cm^{-2}. The sample was loaded on carbon paper with a loading mass of 0.3 mg cm^{-2}.

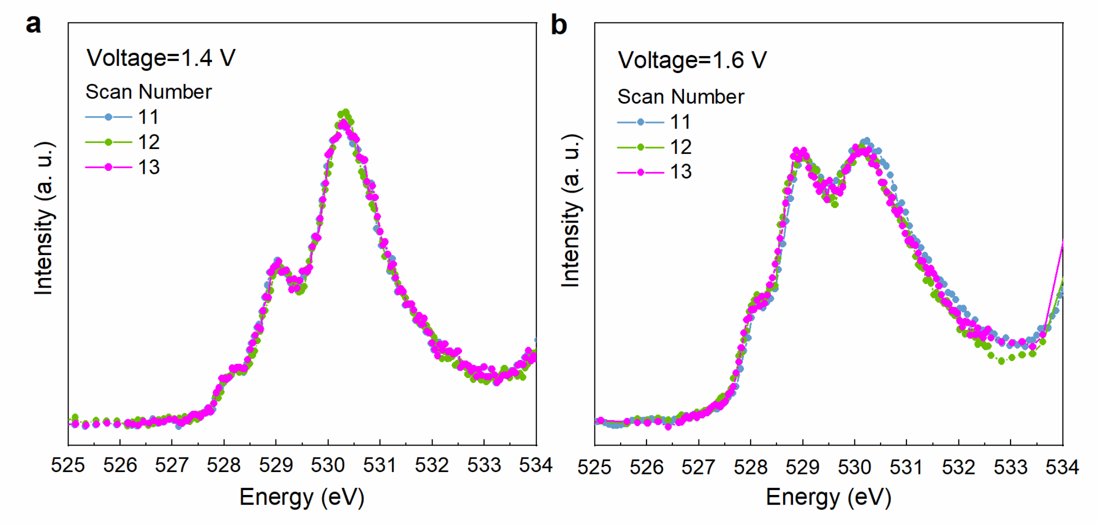

Supplementary Figure 2. *In operando* O-K SXAS spectra of $Li_2Co_2O_4$ with the scan numbers 11th, 12th and 13th under different voltages. **a** 1.4 V, **b** 1.6 V. It indicated a stabilization of the Co valence state after the 11th scan (each scan takes 2 minutes).

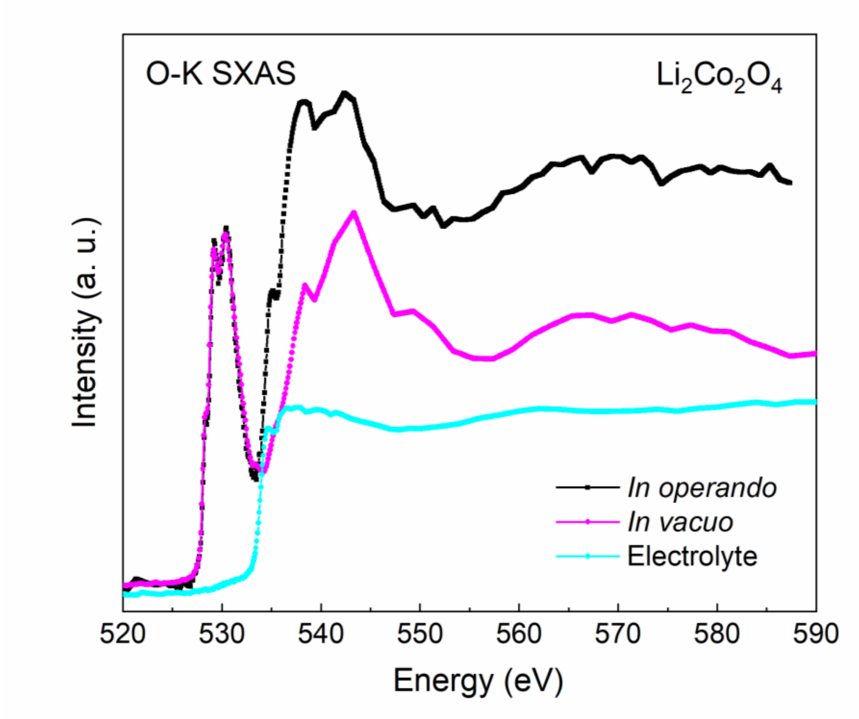

Supplementary Figure 3. Comparison of the O-K SXAS spectra of $Li_2Co_2O_4$ taken *in operando* (black) under an applied voltage of 1.6 V after 20 minutes and *in vacuo* (magenta) after the OER. The high intensity above 533 eV in the *in operando* data can be ascribed to the absorption by the oxygen in the electrolyte as shown by the spectrum of the electrolyte only (cyan). This demonstrates that the oxygen in the electrolyte does not contribute to the pre-edge peaks below 533 eV.

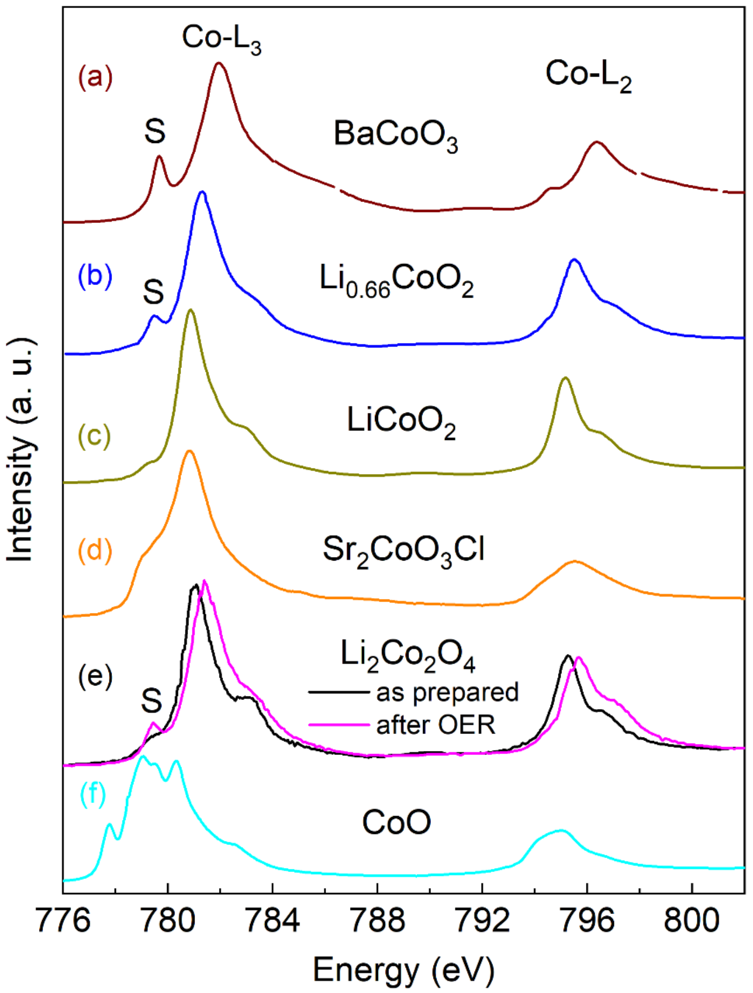

Supplementary Figure 4. Co-$L_{2,3}$ SXAS spectra. **a** $BaCoO_3$ as a low spin Co^{4+} reference (taken from ref. 1), **b** $Li_{0.66}CoO_2$ as a mixed Co^{3+}/Co^{4+} reference (taken from ref. 2), **c** $LiCoO_2$ as a low spin Co^{3+} (taken from ref. 2), **d** Sr_2CoO_3Cl as a high spin Co^{3+} reference with pyramidal local coordination (taken from ref. 3), **e** $Li_2Co_2O_4$ as-prepared (black line, taken under an applied voltage of 0 V) and after OER (magenta line, taken after 20 minutes under an applied voltage of 1.6 V), and **f** CoO as a Co^{2+} reference. Co-$L_{2,3}$ SXAS spectra of $Li_2Co_2O_4$ as-prepared and after OER are taken with TFY mode *in situ* and have been corrected for self-absorption effects. Other spectra are taken with TEY mode.

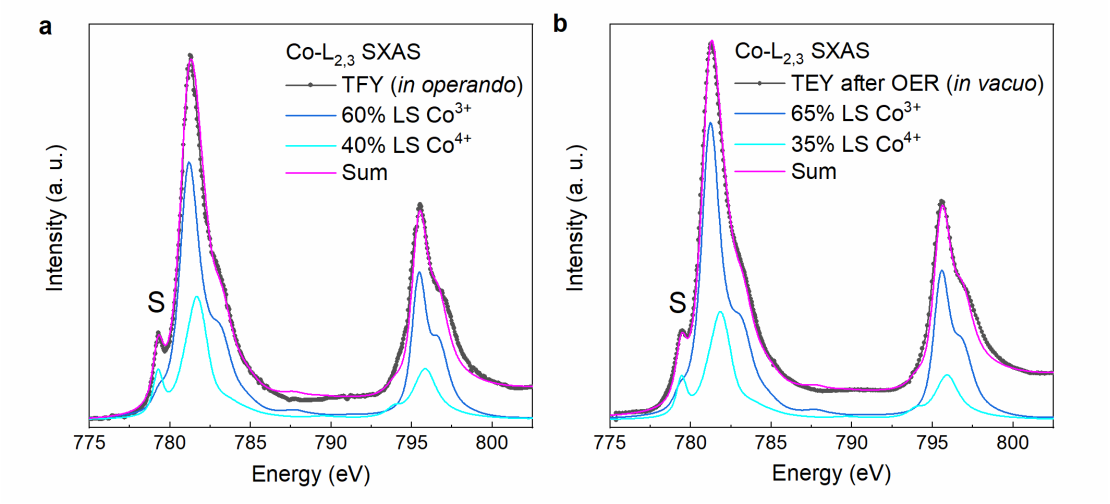

Supplementary Figure 5. Experimental and Theoretical spectra of Co-$L_{2,3}$ SXAS spectra of $Li_2Co_2O_4$. In **a**, experimental Co-$L_{2,3}$ SXAS spectrum of $Li_2Co_2O_4$ was taken *in operando* after 20 minutes under an applied voltage of 1.6 V with the TFY mode (black line). In **b**, experimental data was taken *in vacuo* after the OER with the TEY mode (black line). Theoretical spectra (magenta lines) constructed from a weighted sum of the theoretical simulation for an LS-Co^{3+} spectra (blue lines) and an LS-Co^{4+} spectra (cyan lines) taken from ref. 1.

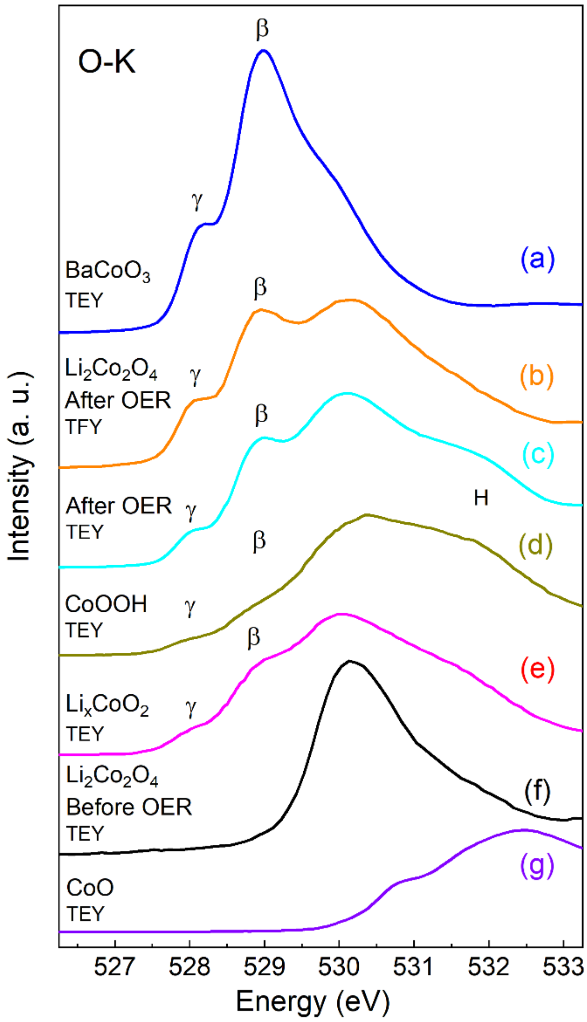

Supplementary Figure 6. O-K SXAS spectra. **a** $BaCoO_3$ (from ref. [4]) as a low spin Co^{4+} reference (TEY mode), **b** $Li_2Co_2O_4$ after OER (TFY mode), **c** $Li_2Co_2O_4$ after OER (TFY mode), **d** CoOOH, **e** Li_xCoO_2 (from ref. [5]) as a mixed Co^{3+}/Co^{4+} reference (TEY mode), **f** $Li_2Co_2O_4$ before OER (TEY mode), and **g** CoO as a Co^{2+} reference (TEY mode). The spectra are arranged from bottom to top to display the spectral changes associated with the increase of the Co valence and the degree of the covalency.

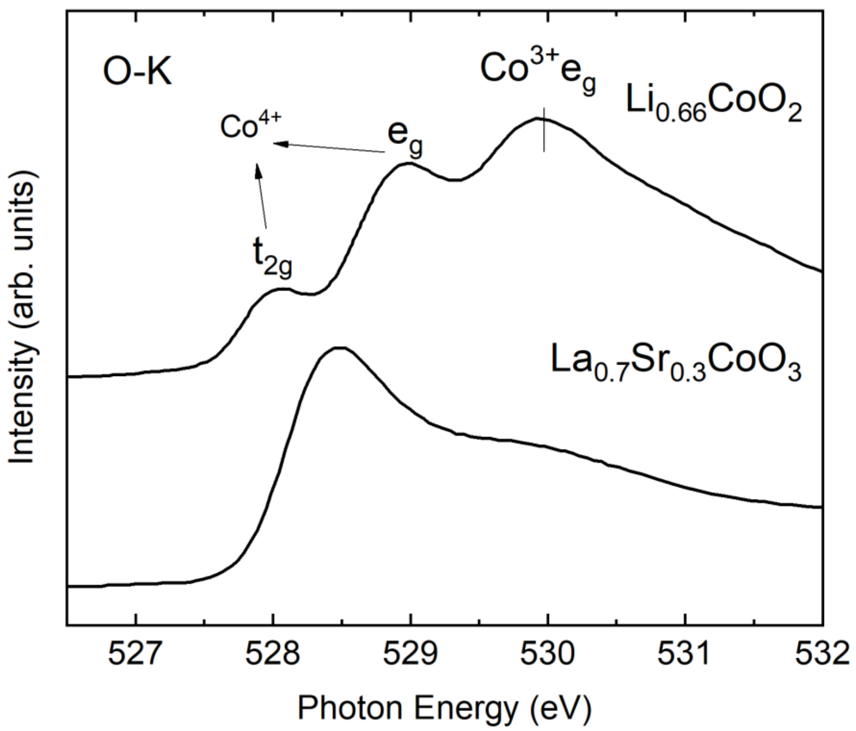

Supplementary Figure 7. The O-K SXAS of $Li_{0.66}CoO_2$ (from ref. 2) and $La_{0.7}Sr_{0.3}CoO_3$ (from ref. 6) for comparison.

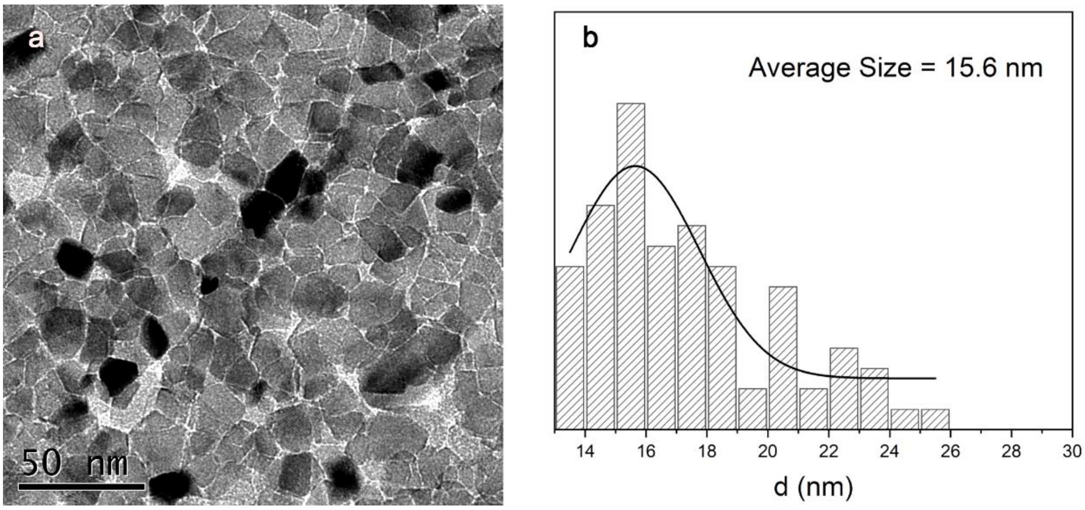

Supplementary Figure 8. Particle size of $Li_2Co_2O_4$**. a** High-resolution transmission electron microscopy (HRTEM) images of the as-prepared $Li_2Co_2O_4$. **b** Particle size distribution determined using the diameters measured on the HRTEM image.

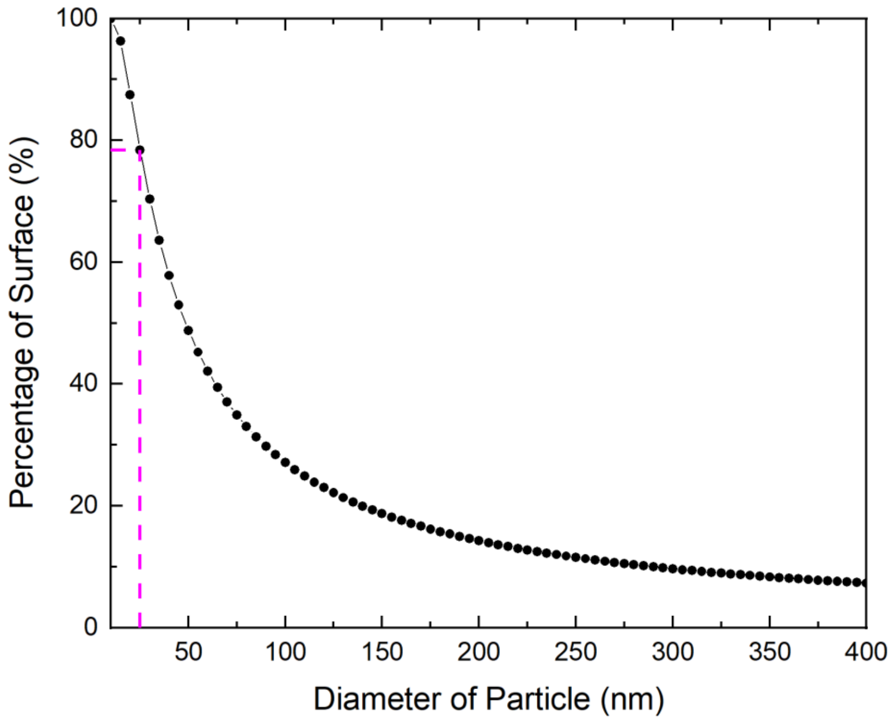

Supplementary Figure 9. The ratio of the active surface region versus bulk contribution to the TFY O-K SXAS spectra as a function of the particle size, assuming a 5 nm thickness of the active surface region and a probing depth of 200 nm.

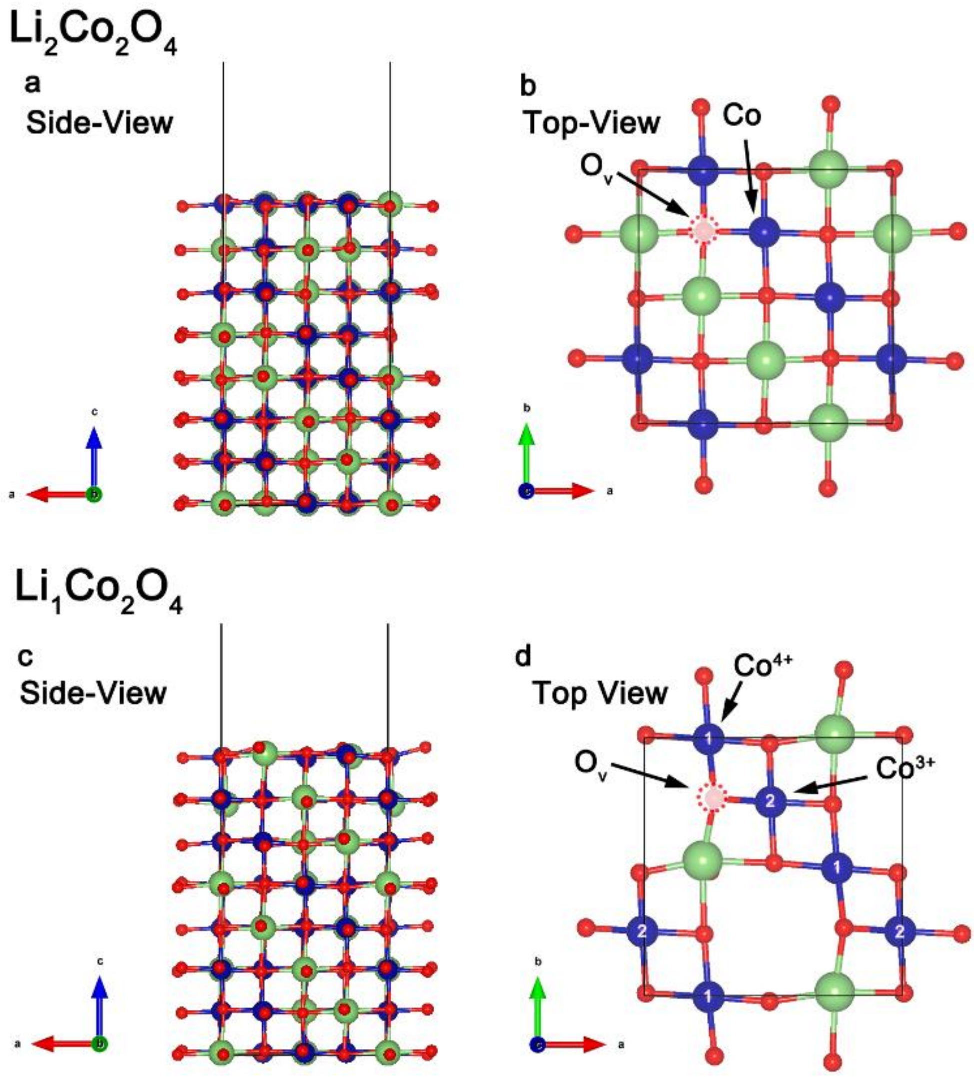

Supplementary Figure 10. The optimized geometry sketches for the (001) surface. **a-b** $Li_2Co_2O_4$, **c-d** $Li_1Co_2O_4$. Blue spheres represent Co, red spheres represent O and green spheres represent Li. For $Li_2Co_2O_4$, only Co^{3+} exists on the surface. For $Li_1Co_2O_4$ sample, there are two different Co sites on the surface: Co^{4+} site and Co^{3+} site, which are labeled as 1 and 2 in **d** respectively. In the MAE and LOV scenarios, the redox behavior take place in the Co sites and oxygen vacancy (Ov) sites, respectively, while in the MLOV both Co and Ov sites take participate in the OER reactions.

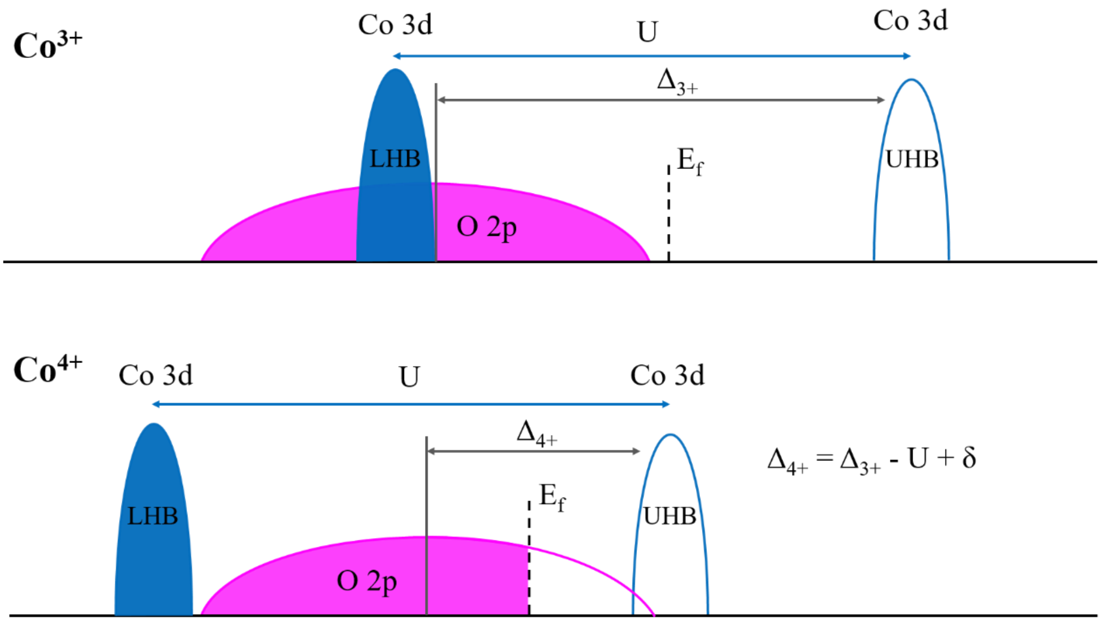

Supplementary Figure 11. The schematic electron removal and addition diagram of Co^{3+} and Co^{4+}-based oxides[7]. Broad O 2p bands are shown in magenta and narrow Co 3d bands in blue. The Fermi level is marked by a dashed vertical line. Considering that the position of the Fermi level E_f is arbitrary in the band gap, we use the broad O 2p band as energy reference. The E_f then shifts towards to the center O 2p band with increasing Co valence.

.

Supplementary Table 1. Composition of the $Li_2Co_2O_4$ sample as-prepared and after the OER reaction under applied voltages of 1.4 V and 1.6 V determined from ICP-OES.

$Li_2Co_2O_4$	The concentrations of metal ions (mg/L)		Composition
	Li	Co	
As-prepared	0.09	0.71	Li : Co = 1.01
1.4V, 4 minutes	0.17	1.65	Li : Co = 0.89
1.4V, 8minutes	0.26	2.63	Li : Co = 0.83
1.4V, 12minutes	0.17	1.87	Li : Co = 0.80
1.4V, 16 minutes	0.27	3.12	Li : Co = 0.74
1.4V, 20 minutes	0.16	1.92	Li : Co = 0.73
1.6V, 4 minutes	0.31	3.91	Li : Co = 0.67
1.6V, 8 minutes	0.14	2.09	Li : Co = 0.58
1.6V, 12 minutes	0.11	1.84	Li : Co = 0.52
1.6V, 16 minutes	0.13	2.10	Li : Co = 0.50
1.6V, 20 minutes	0.16	2.69	Li : Co = 0.50

Supplementary Table 2. DFT-predicted corrections for zero-point energy (eV) and vibrational entropy (eV) of adsorbates.

	ZPE	TS	ZPE-TS	Δ(ZPE-TS)*
**$H_2O(l)$**	0.57	0.67	-0.10	
**$H_2(g)$**	0.28	0.40	-0.12	
**-OH**	0.34	0.10	0.24	0.28
**-O**	0.07	0.06	0.01	-0.01
**-OOH**	0.43	0.28	0.15	0.17
**-OO**	0.13	0.14	-0.02	-0.04
**-OO with Ov**	0.12	0.15	-0.03	-0.05
**-H on lattice O**	0.33	0.10	0.23	0.29

* Δ(ZPE-TS) is with respect to $H_2O(l)$ and $H_2(g)$.

Supplementary Note

Elementary steps and equations which are considered for three scenarios of the reaction mechanism were listed below:

MAE scenario:

$$H_2O + * \rightarrow OH^* + e^- + H^+ \quad (1)$$

$$OH^* \rightarrow O^* + e^- + H^+ \quad (2)$$

$$H_2O + O^* \rightarrow OOH^* + e^- + H^+ \quad (3)$$

$$OOH^* \rightarrow O_2 + e^- + H^+ \quad (4)$$

in which OH*, O* and OOH* represent OH, O and OOH species adsorbed on the metal-site (*) on the surface, respectively.

The Gibbs free energy change for steps a1-a4 can be expressed as

$$\Delta G_{a1} = \Delta G_{OH^*} - eU_{RHE} \quad (5)$$

$$\Delta G_{a2} = \Delta G_{O^*} - \Delta G_{OH^*} - eU_{RHE} \quad (6)$$

$$\Delta G_{a3} = \Delta G_{OOH^*} - \Delta G_{O^*} - eU_{RHE} \quad (7)$$

$$\Delta G_{a4} = -2\Delta g_{H2O}^{exp} - \Delta G_{OOH^*} - eU_{RHE} \quad (8)$$

where U_{RHE} is the potential measured against RHE at standard conditions (T=298.15K, P = 1 bar). Δg_{H2O}^{exp} is the experimental Gibbs free energy of formation of water molecules. ΔG_{OH^*}, ΔG_{O^*} and ΔG_{OOH^*} depend on the adsorption energies of OH*, O* and OOH*, zero point energy (ZPE) and entropy corrections according to $\Delta G_i = \Delta E_i + \Delta ZPE_i - T\Delta S_i$. The adsorption energies are calculated as follows.

$$\Delta E_{OH^*} = E(OH^*) - E(*) - [E(H_2O) - 0.5E(H_2)] \quad (9)$$

$$\Delta E_{O^*} = E(O^*) - E(*) - [E(H_2O) - E(H_2)] \quad (10)$$

$$\Delta E_{OOH^*} = E(OOH^*) - E(*) - [2E(H_2O) - 1.5E(H_2)] \quad (11)$$

Because the bond energy of O_2 is difficult to determine accurately within PBE-

DFT, we estimate the energy of O_2 by $E(O_2) = 2(E(H_2O) - E(H_2) - \Delta g_{H2O}^{exp})$. Thus, the theoretical overpotential is defined as:

$$\eta = \frac{max[\Delta G_{a1}, \Delta G_{a2}, \Delta G_{a3}, \Delta G_{a4}]}{e} - 1.23 \ [V] \tag{12}$$

LOV scenario:

$$OH^{Ov} \rightarrow O^{Ov} + e^- + H^+ \tag{13}$$

$$H_2O + O^{Ov} \rightarrow OOH^{Ov} + e^- + H^+ \tag{14}$$

$$OOH^{Ov} \rightarrow O_V + O_2 + e^- + H^+ \tag{15}$$

$$O_V + H_2O \rightarrow OH^{Ov} + e^- + H^+ \tag{16}$$

in which OH^{Ov}, O^{Ov} and OOH^{Ov} represent OH, O and OOH species adsorbed locate at the lattice oxygen vacancy (O_V) on the surface, respectively. We considered the oxygen vacancy is easy to fill by OH^- in alkaline environment and start the OER reaction from the situation of OH^{Ov}.

The Gibbs free energy change for steps b1-b4 can be expressed as

$$\Delta G_{b1} = \Delta G_{O^{ov}} - \Delta G_{OH^{ov}} - eU_{RHE} \tag{17}$$

$$\Delta G_{b2} = \Delta G_{OOH^{ov}} - \Delta G_{O^{ov}} - eU_{RHE} \tag{18}$$

$$\Delta G_{b3} = -2\Delta g_{H2O}^{exp} - \Delta G_{OOH^{ov}} - eU_{RHE} \tag{19}$$

$$\Delta G_{b4} = \Delta G_{OH^{ov}} - eU_{RHE} \tag{20}$$

$\Delta G_{OH^{ov}}$, $\Delta G_{O^{ov}}$ and $\Delta G_{OOH^{ov}}$ depend on the adsorption energies of OH*, O* and OOH* adsorbed locate at O_V on the surface which can be calculated as follows.

$$\Delta E_{OH^{ov}} = E(OH^{Ov}) - E(O_v) - [E(H_2O) - 0.5E(H_2)] \tag{21}$$

$$\Delta E_{O^{ov}} = E(O^{Ov}) - E(O_v) - [E(H_2O) - E(H_2)] \tag{22}$$

$$\Delta E_{OOH^{ov}} = E(OOH^{Ov}) - E(O_v) - [2E(H_2O) - 1.5E(H_2)] \tag{23}$$

The theoretical overpotential is defined as:

$$\eta = \frac{max[\Delta G_{b1}, \Delta G_{b2}, \Delta G_{b3}, \Delta G_{b4}]}{e} - 1.23 \ [V] \tag{24}$$

MLOV scenario:

$$\mathrm{OH^*} \rightarrow [\mathrm{OO^*} + \mathrm{O_v}] + e^- + \mathrm{H^+} \tag{25}$$

$$\mathrm{H_2O} + [\mathrm{OO^*} + \mathrm{O_v}] \rightarrow \mathrm{O_2} + [* + \mathrm{OH^{Ov}}] + e^- + \mathrm{H^+} \tag{26}$$

$$\mathrm{H_2O} + [* + \mathrm{OH^{Ov}}] \rightarrow [\mathrm{OH^*} + \mathrm{OH^{Ov}}] + e^- + \mathrm{H^+} \tag{27}$$

$$[\mathrm{OH^*} + \mathrm{OH^{Ov}}] \rightarrow \mathrm{OH^*} + e^- + \mathrm{H^+} \tag{28}$$

The Gibbs free energy change for steps c1-c4 can be expressed as

$$\Delta \mathrm{G_{c1}} = \Delta \mathrm{G_{OO^*+Ov}} - \Delta \mathrm{G_{OH^*}} - e\mathrm{U_{RHE}} \tag{29}$$

$$\Delta \mathrm{G_{c2}} = -2\Delta \mathrm{g_{H2O}^{exp}} + \Delta \mathrm{G_{*+OH^{Ov}}} - \Delta \mathrm{G_{OO^*+Ov}} - e\mathrm{U_{RHE}} \tag{30}$$

$$\Delta \mathrm{G_{c3}} = \Delta \mathrm{G_{OH^*+OH^{Ov}}} - \Delta \mathrm{G_{*+OH^{Ov}}} - e\mathrm{U_{RHE}} \tag{31}$$

$$\Delta \mathrm{G_{c4}} = \Delta \mathrm{G_{OH^*}} - \Delta \mathrm{G_{OH^*+OH^{Ov}}} - e\mathrm{U_{RHE}} \tag{32}$$

$\Delta \mathrm{G_{OO^*+Ov}}$, $\Delta \mathrm{G_{OH^*}}$, $\Delta \mathrm{G_{OH^*+OH^{Ov}}}$ and $\Delta \mathrm{G_{*+OH^{Ov}}}$ depend on the corresponding adsorption energies as follows.

$$\Delta \mathrm{E_{OH^*}} = \mathrm{E(OH^*)} - \mathrm{E(*)} - [\mathrm{E(H_2O)} - 0.5\mathrm{E(H_2)}] \tag{33}$$

$$\Delta \mathrm{E_{OO^*+Ov}} = \mathrm{E(OO^*} + \mathrm{O_v)} - \mathrm{E(*)} - [\mathrm{E(H_2O)} - \mathrm{E(H_2)}] \tag{34}$$

$$\Delta \mathrm{E_{*+OH^{Ov}}} = \mathrm{E(* + OH^{Ov})} - \mathrm{E(*)} - [0.5\mathrm{E(H_2)}] \tag{35}$$

$$\Delta \mathrm{E_{OH^*+OH^{Ov}}} = \mathrm{E(OH^*} + \mathrm{OH^{Ov})} - \mathrm{E(*)} - \mathrm{E(H_2O)} \tag{36}$$

The theoretical overpotential is defined as:

$$\eta = \frac{\max[\Delta \mathrm{G_{c1}}, \Delta \mathrm{G_{c2}}, \Delta \mathrm{G_{c3}}, \Delta \mathrm{G_{c4}}]}{\mathrm{e}} - 1.23 \ \ [\mathrm{V}] \tag{37}$$

Supplementary references

1. Lin, H. J. et. al. Local orbital occupation and energy levels of Co in Na_xCoO_2: A soft x-ray absorption study. *Phys. Rev. B* **81**, 115138 (2010).
2. Mizokawa, T. et. al. Role of oxygen holes in Li_xCoO_2 revealed by soft X-ray spectroscopy. *Phys. Rev. Lett.* **111**, 056404 (2013).
3. Hu, Z. et. al. Different look at the spin state of Co^{3+} Ions in a CoO_5 pyramidal coordination. *Phys. Rev. Lett.* **92**, 207402 (2004).
4. Y. Chin et. al. Spin-orbit coupling and crystal-field distortions for a low-spin $3d^5$ state in $BaCoO_3$. arXiv:1905.09549, (2019).
5. Yoon, W.-S. et. al. Oxygen contribution on Li-ion intercalation−deintercalation in $LiCoO_2$ investigated by O K-edge and Co L-edge X-ray absorption spectroscopy. *J. Phys. Chem. B* **106**, 2526-2532 (2002).
6. Merz, M. et. al. X-ray absorption and magnetic circular dichroism of $LaCoO_3$, $La_{0.7}Ce_{0.3}CoO_3$, and $La_{0.7}Sr_{0.3}CoO_3$ films: Evidence for cobalt-valence-dependent magnetism. *Phys. Rev. B* **82**, 174416 (2010).
7. Zaanen, J., Sawatzky, G. A. & Allen, J. W., Band gaps and electronic structure of transition-metal compounds. *Phys. Rev. Lett.* **55**, 418-421 (1985).
